# Stable Photosymbiotic Relationship under CO_2_-Induced Acidification in the Acoel Worm *Symsagittifera Roscoffensis*

**DOI:** 10.1371/journal.pone.0029568

**Published:** 2012-01-09

**Authors:** Sam Dupont, Aurélie Moya, Xavier Bailly

**Affiliations:** 1 Department of Marine Ecology, University of Gothenburg, Fiskebäckskil, Sweden; 2 INSU-CNRS & UPMC University of Paris 06, Laboratoire d'Océanographie de Villefranche, Villefranche-sur-mer, France; 3 ARC Centre of Excellence for Coral Reef Studies, James Cook University, Townsville, Australia; 4 Université Pierre et Marie Curie - CNRS, FR2424, Station Biologique de Roscoff, Roscoff, France; US Dept. of Agriculture – Agricultural Research Service (USDA-ARS), United States of America

## Abstract

As a consequence of anthropogenic CO_2_ emissions, oceans are becoming more acidic, a phenomenon known as ocean acidification. Many marine species predicted to be sensitive to this stressor are photosymbiotic, including corals and foraminifera. However, the direct impact of ocean acidification on the relationship between the photosynthetic and nonphotosynthetic organism remains unclear and is complicated by other physiological processes known to be sensitive to ocean acidification (e.g. calcification and feeding). We have studied the impact of extreme pH decrease/*p*CO_2_ increase on the complete life cycle of the photosymbiotic, non-calcifying and pure autotrophic acoel worm, *Symsagittifera roscoffensis*. Our results show that this species is resistant to high *p*CO_2_ with no negative or even positive effects on fitness (survival, growth, fertility) and/or photosymbiotic relationship till *p*CO_2_ up to 54 K µatm. Some sub-lethal bleaching is only observed at *p*CO_2_ up to 270 K µatm when seawater is saturated by CO_2_. This indicates that photosymbiosis can be resistant to high *p*CO_2_. If such a finding would be confirmed in other photosymbiotic species, we could then hypothesize that negative impact of high *p*CO_2_ observed on other photosymbiotic species such as corals and foraminifera could occur through indirect impacts at other levels (calcification, feeding).

## Introduction

Photosymbiosis, i.e. symbiosis between photosynthetic and nonphotosynthetic organisms, is widespread and underlies a wide range of ecologically and biogeochemically significant processes. In the oceans, this includes metazoans with the emblematic calcifying corals (symbiosis between a cnidaria and a dinoflagellate) that build globally important coral reefs as well as many protists (unicellular eukaryotes) such as foraminiferans, acantharans and polycystines (Rhizaria) or ciliates and dinoflagelates (Alveolates) or Mayorella and Thecamoeba (Amoebozoa) [1]. These photosymbioses (some species having unicellular algae as endosymbionts, from diverse lineages such as the green algae, red algae, golden algae, diatoms, and dinoflagellates) are important contributors to flux in the deep ocean. Many of these taxa are calcifiers and the presence of the algae is required for the high rate of calcification (“light enhanced calcification”, [2]–[4]). The symbiosis is regulated by intrinsic and extrinsic parameters (e.g. environmental stress) and can break down, a phenomenon known as bleaching, under certain conditions including increased temperature and *p*CO_2_ (e.g. [5] for corals and [6] for Foraminifera). However, the factors that allow initiation and maintenance of a stable relationship remain largely unclear and to make any prediction on the impact of near-future rapid predicted environmental changes on photosymbiotic species it is critical to understand how host-symbiont relationship could be affected by these changes [7].

As a consequence of human activity, atmospheric CO_2_ concentration has increased from a preindustrial value of about 280 µatm to a current value of 380 µatm. Models predict that it could reach value as high as 1200 µatm by the end of the century. The world's oceans represent a major sink and half of this excess of CO_2_ is being absorbed by the ocean. The increase in partial pressure of CO_2_ is predicted to induce a pH decrease by 0.4 units by 2100 corresponding to seawater two times more acidic, a phenomenon known as ocean acidification ([8]).

Ocean acidification research is a relatively new field and a good deal of effort has been invested in studying predicted sensitive taxa, in particular the marine calcifiers (see [9]for review). Among these, corals have received a considerable amount of attention. For example, studies have shown dissolution of coral skeletons (e.g. [10]) and reduced rate of reef calcification [11] under low pH conditions. It was also proposed that acidification may affect the relationship between corals and their symbiotic algae and the productivity of the association. Thus, high CO_2_ impacts corals and crustose coralline algae more strongly on bleaching (large scale disintegration of the coral-algae symbiosis) and productivity than on calcification [12]. On the other hand, Fine and Tchernov (2007) showed that the symbiosis could be maintained in skeleton free coral (solitary coral polyps following skeleton dissolution) kept for over a year under very high CO_2_ conditions (ΔpH = −0.8). A recent study on the impact of acidified water on early stages of scleractinian corals showed that the non-calcifying planula larval stage exposed to low pH (ΔpH = 0.7) had the same mortality rate as in control conditions. The calcifying polyp had a decreased growth and algal infection rates under low pH conditions [13]. However, it is unclear if delayed establishment of symbiosis is a direct impact of the low pH conditions or just an indirect effect on growth rate (see [14] for further discussion on the problem of data interpretation when growth rates are impacted). It was also suggested that foraminifera may be particularly vulnerable to ocean acidification, showing shell mass decrease and bleaching [1].

As previously mentioned, a lot of effort was invested in studying the impact of possible near-future ocean acidification on predicted sensitive species. Little research focus is currently placed on those organisms/taxa that might be less vulnerable to the anticipated changes in ocean chemistry; this is unfortunate, as the comparison of more vulnerable to more tolerant physiotypes could provide us with those physiological traits that are crucial for ecological success in a future ocean [15]. Moreover, invertebrates represent 95% of all animal species and are divided into 38 phyla, 35 having marine representatives and 18 being exclusively marine [16]. There is a high pressure from society to provide information on impact of near-future climate changes on marine ecosystem and it is then of vital importance to understand how ocean acidification may impact marine invertebrates and their related ecosystems where they often play key ecological functions. Today, we only have information on the impact of ocean acidification on 14 invertebrate phyla with most of the research being focused on cnidarians, crustaceans, mollusks and echinoderms. It is then of crucial importance to gather information on other neglected phyla.

One of these neglected groups are acoel worms. Acoels have mainly been described as coastal organisms. However, they are suspected to be ecologically significant in open oceans and may represent an unexpected source for primary productivity [1]. Moreover, photosymbiosis is also observed between the acoel worm *Symsagittifera roscoffensis* and the green algal *Tetraselmis convolutae*. This basal bilaterian or deuterostome (see [17] for discussion regarding its taxonomic position) is a small “simple” gregarious free living marine organism up to 4 mm in length. As an adult it can contain up to 40,000 algal cells [18]. Despite the presence of a mouth and in striking contrast with corals, the adults do not ingest food but grow completely autotrophically [19]. *S. roscoffensis* is endemic to the tidal zone of the European coast of the Atlantic Ocean and English Channel from Portugal to Wales. They form colonies that can reach millions of individuals per square meter on sandy beaches with run-off water. They live in the sandy sediment during high tide to avoid dispersal by waves and migrate to the sand surface during low tide/daytime to be exposed to sunlight that favours photosynthetic activity of the symbiotic algae. This association is extremely efficient and an annual primary productivity of 872 gC.m^−2^ has been recorded, approaching estimates for coral reefs [18]. It is a hermaphroditic species and during the breeding season (September to June), it can lay up to 30 eggs per individual. Eggs are spawned within the sediment and enclosed in a cocoon. Embryonic development lasts 5 to 6 days at 15°C before hatching of a colourless free-living aposymbiotic juvenile. Without initiation of the symbiosis, juveniles do not survive for more than 20 days. Generation time is around 45 to 60 days (see [17] for complete description of the species).

*S. roscoffensis* provides an interesting alternative to coral and foraminifera for investigation of the impact of environmental perturbation on photosymbiosis. As a tidal species, it is potentially very resistant to low pH/high CO_2_ and it allows the investigation of direct impacts of environmental stress on the photosymbiotic relationship without interaction with other processes potentially impacted by low pH such as calcification and/or heterotrophic feeding.

In this paper, the impact of a range of pH/pCO_2_ (from 400 µatm to super-saturated waters) on the acoel worm *S. roscoffensis* was investigated taking into account the complete life-cycle.

## Results

**Figure 1:**
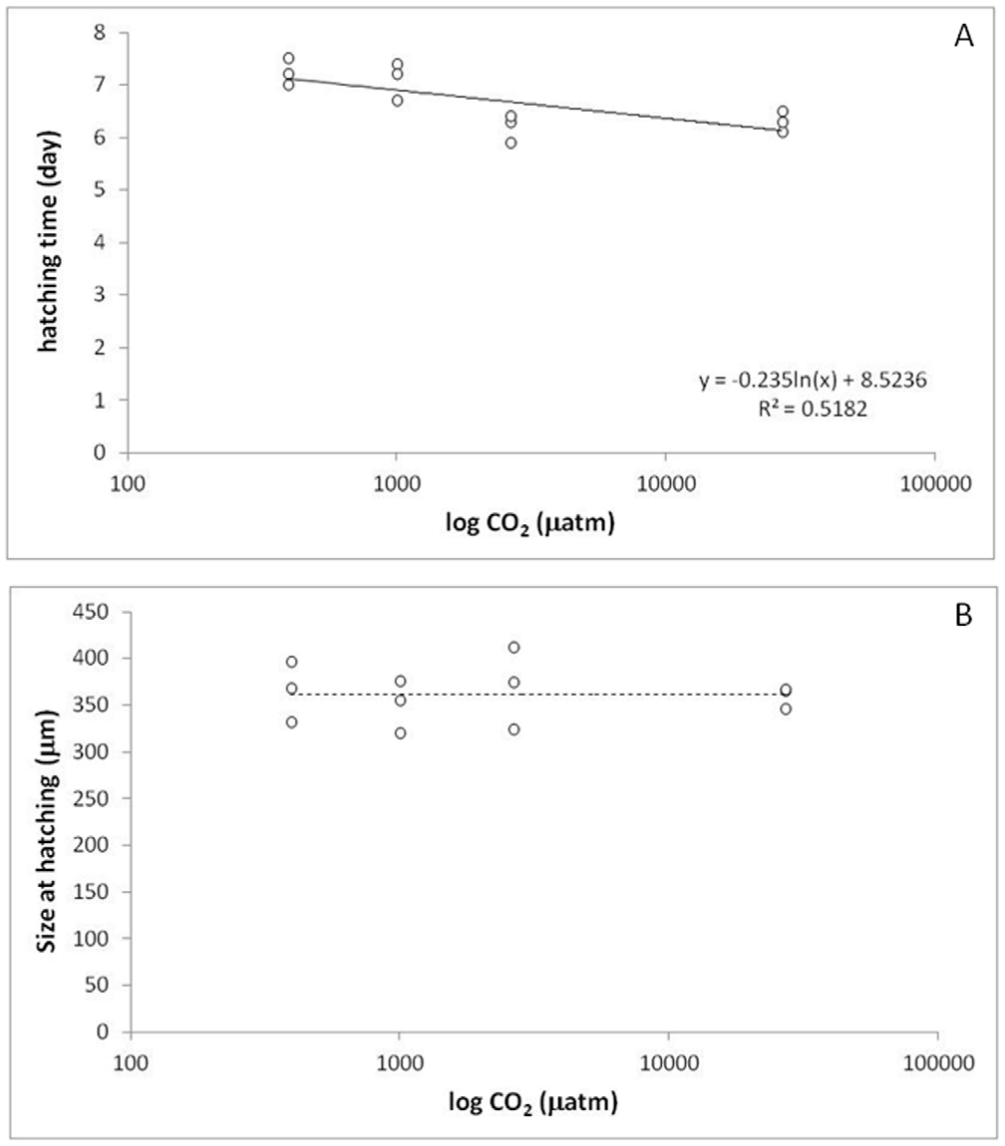
Impact of a range of *p*CO_2_ on: A. median hatching time; B. size of the juvenile at hatching (Experiment 1). A significant logarithmic relationships was observed between *p*CO_2_ and hatching time of cocoons (F = 10.76, p<0.008) but not between *p*CO_2_ and juvenile size at hatching time within the range of tested *p*CO_2_ (F = 0.01, p<0.97).

A significant logarithmic relationships was observed between *p*CO_2_ and hatching time of cocoons (F = 10.76, p<0.008; Figure 1A) but not between *p*CO_2_ and juvenile size at hatching time (362 µm) within the range of tested *p*CO_2_ (F = 0.01, p<0.97; Figure 1B). For example, when raised in the highest *p*CO_2_ environment (27 k µatm), cocoons hatched 13% faster (41 day faster) compared to the lowest (0.4 k µatm).

**Figure 2:**
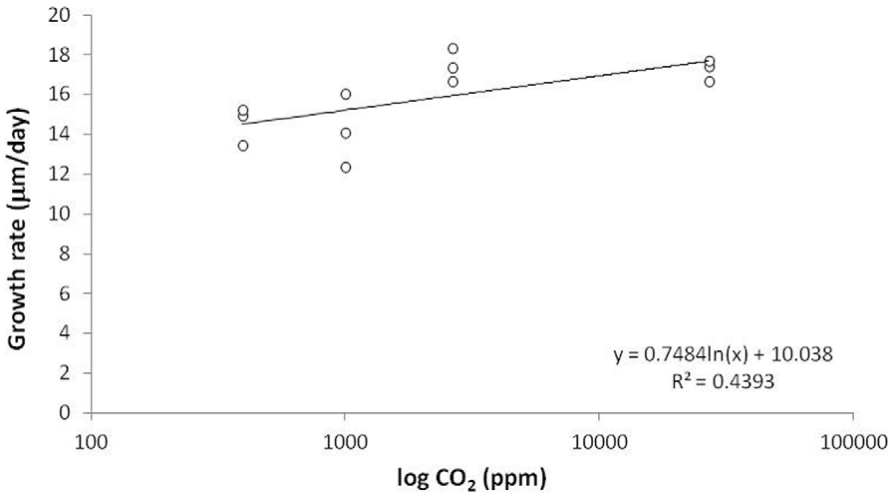
Impact of a range of *p*CO_2_ on the growth rate of newly hatched juveniles in presence of their photosymbiotic algae (9 days exposure; Experiment 3). A significant logarithmic relationships was observed between *p*CO_2_ and juvenile growth rate (F = 7.84, p<0.019), the growth rate being 19% faster in the highest test *p*CO_2_ (27 k µatm) compared to the lowest (0.4 k µatm).

Even when exposed to high *p*CO_2_ as much as 27 k µatm, no mortality was observed on newly hatched juveniles for up to 5 days in absence of the symbiotic algae (experiment 2) and up to 9 days in the presence of the symbiotic algae (experiment 3). Moreover, after 9 days, the presence of symbiotic algae was observed in all exposed juveniles. A significant logarithmic relationships was observed between *p*CO_2_ and juvenile growth rate (F = 7.84, p<0.019; Figure 2), the growth rate being 19% faster in the highest test *p*CO_2_ (27 k µatm) compared to the lowest (0.4 k µatm).

**Figure 3:**
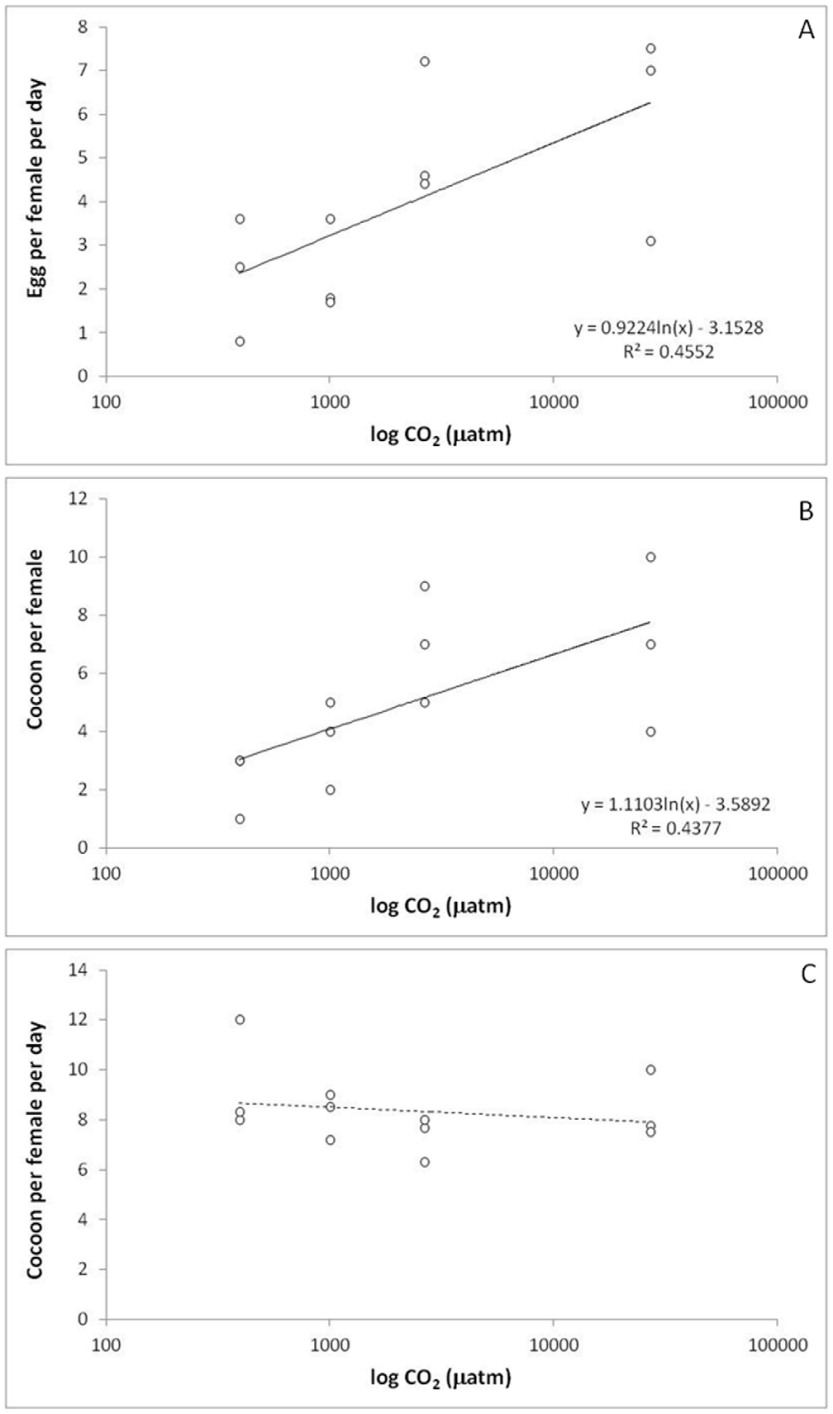
Impact of a range of *p*CO_2_ on: A. number of eggs produced per female; B. number of eggs per cocoon; C. number of cocoon produced per female (experiment 4). The number of eggs produced per female was 3 times higher in high compared to low *p*CO_2_ (27 k vs 0.4 k µatm) and a significant logarithmic relationship was observed between the two parameters (F = 8.36, p<0.016; Figure 3A). This was the consequence of an increased number of cocoon produced per female (significant logarithmic relationship, F = 7.79, p<0.02; Figure 3B) with no effect on the number of eggs per cocoon (non significant logarithmic relationship, F = 0.43, p<0.53; Figure 3C).

The number of eggs produced per female was 3 times higher in high compared to low *p*CO2 (27 k vs 0.4 k µatm) and a significant logarithmic relationship was observed between the two parameters (F = 8.36, p<0.016; Figure 3A). This was the consequence of an increased number of cocoon produced per female (significant logarithmic relationship, F = 7.79, p<0.02; Figure 3B) with no effect on the number of eggs per cocoon (non significant logarithmic relationship, F = 0.43, p<0.53; Figure 3C).

**Figure 4:**
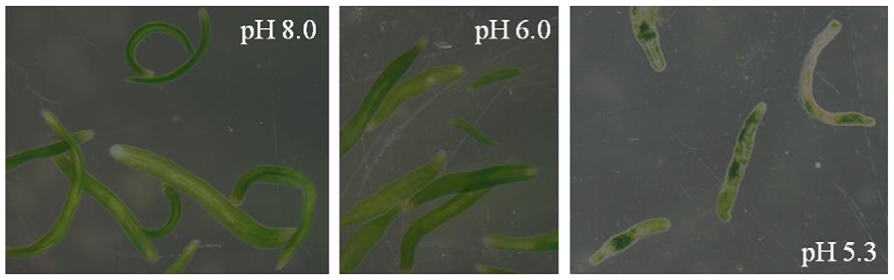
*Symsagittifera roscoffensis* after a 24 h exposure to seawater with different pH (experiment 5). Animals in the lower pH started to bleach.

We tested a range of *p*CO_2_ low*p*CO_2_ = 0.4 k µatm) to seawater saturated with CO_2_ (*p*CO_2_ = 240 k µatm) on adults (experiment 5). After 3 days, no mortality was observed in any of the tested *p*CO_2_ and *S. roscoffensis* presented a normal behavior and general morphology up to *p*CO_2_ = 47 k µatm). At higher *p*CO_2_, after 24 hours we observed the beginning of expulsion of the symbiotic algae (Figure 4).

## Discussion

**Figure 5:**
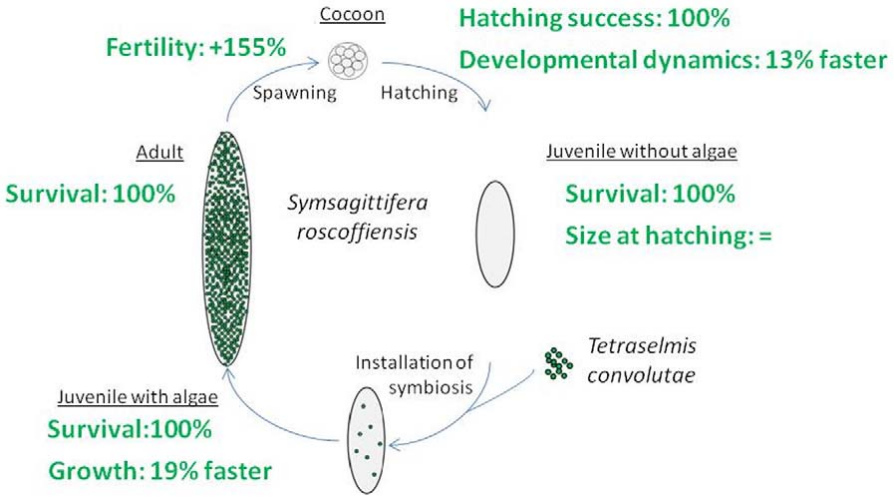
Summary of the impact of low *p*CO_2_ (27063 µatm) on *S. roscoffensis* life cycle.

*S. roscoffensis* appears to be resistant and even benefits from high *p*CO_2_ environment. Under high *p*CO_2_ (up to 27 k µatm), many parameters appear to be positively impacted, for example faster growth and development and a higher fertility with no negative effect on other parameters such as survival (Figure 5). Negative effect (sublethal bleaching) was only observed under extreme high *p*CO_2_ environment (240 k µatm). Exposure of the symbiotic acoel to increased CO_2_ concentration has traditionally been used as a method to induce expulsion of the algae [20]. When exposed to extreme acidic conditions such as seawater saturated of CO_2_, *S. roscoffensis* changes its behavior and rapidly starts expulsion of the symbiotic *T. convolutae*. After 24 h, the acoel appears to be whitish with some remaining green spots and can survive for several days in this condition [20]. However, we have shown that this effect only appear when seawater is super-saturated with CO_2_ (240 k µatm).

This resistance to high *p*CO_2_ was hypothesized within the range of environmental conditions naturally experienced by this species. However, the species resilience and the maintenance of an efficient photo/symbiosis under *p*CO2 environment up to 27 k µatm is more surprising and contrasts with observations made on other photosymbiotic models such as coral and foraminifera [5], [6].

### Ecological perspectives

A review of the ocean acidification literature reveals that the impact of high *p*CO_2_ appears to be species-specific, even in closely related taxa, and differences in tolerance can be observed within the same species (e.g. see [21]). It is of crucial importance to understand the origin of observed differences and understand the mechanistic principles behind high *p*CO_2_ resilience [15]. It is tempting to hypothesize that species naturally exposed to variable *p*CO_2_ conditions may be pre-adapted to high *p*CO_2_ [21], an idea well documented for temperature stress (e.g. [22]). The thermal niche is a good predictor of latitudinal range supporting both “Brown's hypothesis” (species' fundamental niche determines the size of its geographical range) and “Environmental Variability Hypothesis” (species with greater latitudinal extents have broader physiological limits). Local and temporal variability can shape species ability to cope with any given stressor. For example, it was recently shown that the macrobenthic community in the Kiel Fjord experiencing seasonal upwelling of CO_2_ rich waters (>2300 µatm), is dominated by calcifying invertebrates [23]. This indicates that many calcifying keystone species predicted to be sensitive to near-future ocean acidification (e.g. mussels) may be able to cope with surface ocean *p*CO_2_ values projected for the end of this century when living in a highly variable environment.

Another example is intertidal organisms. They have become adapted to survive the rapid and significant changes in temperature, salinity, humidity, pH, dissolved oxygen, and food supply that occur on a daily basis due to circatidal rhythm. As a tidal species, *S. roscoffensis* is naturally exposed to large environmental variations. It can survive temperatures ranging between 7 and 20°C reflecting the annual temperature range in its habitat. The tidal zone is also known to be extremely variable for other environmental parameters. For example, important diurnal/tidal changes were reported in rockpool in parameters such as pH ([7.29–10.16], for Roscoff, [24]) caused by the community respiration and photosynthesis. *In situ* measurement of pH in the beach run-off water where colonies of *S. roscoffensis* revealed an average pH of 7.5 units, similar to that of seawater at high tide and indicating that run-off water was seawater from the previous tide [18]. The species can be exposed to pH as low as 7.3 for short period of time and is then naturally exposed to *p*CO_2_ ranging from 0.4 to 4 k µatm. The observed resistance to high *p*CO_2_ in this range is expected. However, resistance and even benefit out of the natural range (up to 27 k µatm) is more surprising.

*S. roscoffensis* could be exposed to *p*CO_2_ ranging between 1 and 10 k µatm in the near-future oceans. These values are still in the tolerance range of the species and it is tempting to conclude that the *S. roscoffensis* and its photosymbiosis with *T. convolutae* should not be negatively impacted by near-future ocean acidification. However, it is important to remember that these results are based on a perturbation experiment only considering one environmental parameter (*p*CO_2_/pH), ignoring ecological interactions and long term/multi-generation effects. As a consequence, we may under- or over-estimate the consequences of near-future environmental conditions on the fitness of *S. roscoffensis*.

This also highlights the importance to investigate the natural environment experienced by a given species to define a proper control when designing an experiment. Many perturbation experiments testing the impact of ocean acidification are only considering 2 *p*CO_2_ based on predicted changes in global surface oceans (0.4 vs 1 k µatm). However, it is often overlooked that many (local) marine habitats are naturally exposed to high pH variations and that the predicted changes will occur on top of these variations. This includes upwelling zones [23], [25], coastal areas [26], esturarine and CO_2_ vents [27].

### Physiological perspective

Most of these effects could be explained by a general increase in metabolism and/or energy production. Several studies shown an increased metabolism under high *p*CO_2_ sometime associated with an increased growth (e.g. [28], [29], [30]). Moreover, autotrophic organisms are believed to benefit from high *p*CO_2_ when lacking efficient carbon-concentrating mechanisms (CCM, but see [31]). More energy available associated to an increase in metabolism may then explain the increase in growth (more energy available for growth) and fertility (more energy to be invested in gonadal production).

The negative effects observed on coral species are often associated with calcification (e.g. [13], [32]). However, other parameters may play an equally important role. For example, a direct impact of increased CO_2_ on the coral symbiotic algae photosynthetic mechanism has been proposed suggesting that it may be a consequence of an inhibitory effect on the photoprotective mechanism leading to reduced photorespiration [12]. This hypothesis is not supported by our results showing resilience to high *p*CO_2_ including the maintenance of the photosymbiotic relationship down to *p*CO_2_ 47 k µatm. If such a finding would be confirmed in other photosymbiotic species, we could then hypothesize that negative impact of high *p*CO_2_ could occur through affects at other levels. For example, *p*CO_2_ could affect the ability of the polyp to capture food as it has been shown for echinoderm larvae (e.g. brittlestars, [33]). Organisms that carry out acquired phototrophy are usually mixotrophs (e.g. corals) and the degree to which they depend on phototrophy is variable [1]. Understanding the mechanism that underlies the observed impacts of high *p*CO_2_ on corals is complicated by the complexity of taxa that calcify and combine autotrophy and heterotrophy [34]. In contrast *S. roscoffensis* which is non-calcifying and solely an autotrophic species (see [17] for review) it should be far easier to tease apart these mechanisms. Greater knowledge of physiological interactions between photosynthesis and other processes (including calcification in corals and foraminifera) under high CO_2_ conditions is needed [7], [35], [36]. In this perspective, acoel worms appear as excellent complementary models to foraminifera and corals to enable understanding of the mechanisms of potential resistance to near-future ocean acidification and establishment of the symbiosis.

To what extent this extreme resistance is a direct consequence of the photosymbiosis is very hard to determine without solid physiological evidence. However, some other works allow hypothesizing that extreme resistance (e.g. oxidative stress, solar irradiance, chemical stress, etc.) may be a consequence of symbiosis (see [37]). On the other hand, one could imagine that other and symbiosis-unrelated resistance mechanisms such as strong and efficient pH regulation may evolve in such a harsh environment. However, it is interesting to notice that other photosymbiotic species such as the anemone *Anemonia viridis*, is the only Cnidarian present in acidic waters at site of Ischia [27]. It was hypothesized that this species may benefit from increased *p*CO_2_ for photosynthesis of its endosymbiotic dinoflagellates [27]. This may seems counter intuitive since *A. viridis* possesses CCM but it was previously shown that CO_2_ diffusion can be preferred to the costly bicarbonate uptake (in coral/dinoflagelate relationship, [38]).

In *S. roscoffensis*, the host provide favorable conditions to the photosymbiotic entity while the the algae is a source of nutrient. For example, photosynthetic activity of the algae contributes to host growth by provision of photosynthetates such as mannitol, which are source for the synthesis/release of amino acids for the worm and lactic acid representing a source of nutrients for the host. On the other hand, endogenous uric acid produced by the worm is an important nitrogen source for the algae [39]. The host being purely autotrophic, the stability of the symbiosis is then vital. More experiment are now needed to understand the mechanisms behind this extreme stability under high *p*CO_2_ conditions and what cause the disruption of the photosymbiotic relationship under extreme high *p*CO_2_ (240 k µatm). On the other hand, more information is also needed on the direct impact of high *p*CO_2_ on the free algal symbiont. If affected, this may constitute a bottleneck for the host fitness with potential dramatic consequence on survival.

## Materials and Methods

### Species studied

*S. roscoffensis* and its symbiont *T. convolutae* are both available in culture at the Station Biologique de Roscoff (see [17] for more information). In summary, adult gravid *S. roscoffensis* specimens were collected at low tide in Brittany (Carentec, France) and stored in a thermoconstant chamber at the Station Biologique de Roscoff at 15°C. No legal or ethic permits are required for field collections and experimental studies on Acoel worms. However, individuals were anaesthetized by 7% MgCl_2_ solution before any observation. After 4 days under light intensity of 300 µE with a circadian rhythm of 10 hours light and 14 dark, adults start to produce translucent cocoons containing embryos. Larval development lasts 5 to 6 days before hatching of colourless aposymbiotic juveniles. The prasinophyte *T. convolutae*, the algal photosymbiont of *S. roscoffensis*, was isolated in the Station Biologique de Roscoff by Ian Probert directly from the host. The algal strain is registered in the Roscoff Culture Collection (http://www.sb-roscoff.fr/Phyto/RCC/) under numbers 1563 and 1564 and is available in culture.

### Seawater

Natural aerated and filtered (0.5 µm) seawaters (salinity 34 ppt) with different pH/pCO_2_ were prepared using a computerized control system (AquaMedic) that regulated pH, by the addition of pure gaseous CO_2_ directly into the experimental tank. The pH values of the pH-computer were adjusted from measurements of pH on the total scale (pH_TS_). Thus was measured in each experimental beaker following best practiced for ocean acidification research [40] and using a pH meter (Metrohm, 826 pH mobile) with a glass electrode (Metrohm, electrode plus) calibrated on the total scale using Tris/HCl and 2-aminopyridine/HCl buffer solutions with a salinity of 38 and prepared according to Dickson *et al.* [41]. *p*CO_2_ was calculated from pH_TS_ and alkalinity (TA = 2330 mEq kg^−1^) using sea water CO2 (SWCO2) with dissociation constants from Mehrbach et al. [42] refitted by Dickson & Millero [43]. The treatments were: pH_TS_ = 8.06 (*p*CO_2_ = 391 µatm), pH_TS_ 7.7 (*p*CO_2_ = 991 µatm), pH_TS_ 7.3 (*p*CO_2_ = 2,621 µatm), pH_TS_ 6.3 (*p*CO_2_ = 27,063 µatm), pH_TS_ 6.0 (*p*CO_2_ = 54,112 µatm) and pH_TS_ 5.3 (*p*CO_2_ = 272,261 µatm). Daily seawater changes allowed minimizing the seawater chemistry changes due to biological activity with no pH_TS_ changes higher than 0.06 units.

### Experimental design

The impact of CO_2_-driven acidification is known to be life-cycle stage and process specific [44]. Therefore in order to fully understand a species response, it is essential to include all life-cycle stage and different key fitness-related processes. Our experiment was designed to include all *S. roscoffensis* life-cycle stages. The same experimental set-up was used for all the experiments. Individuals (cocoon, newly hatched juveniles or adults) were placed in 12 to 18 plastic dishes (4–6 *p*CO_2_ ×3 replicates) containing 5 ml of experimental seawater. The dishes were then sealed to avoid any gas exchange and seawater was changed daily. Experimental dishes were kept in a thermoconstant chamber at 15°C under a light intensity of 300 µE with a circadian cycle of 12 hours of light and 12 of dark.

#### Experiment 1: Impact on development

For each replicate, ten cocoons containing eggs at the two cells stage were counted from the culturing facilities and exposed to different *p*CO_2_ (from 390 to 27000 µatm) for up to 9 days. The number of hatched cocoons was counted daily. The median hatching time was estimated as the time to reach 50% of hatching [45]. Newly hatched juveniles were anesthetized using 7% MgCl_2_ in FSW and measured (total length).

#### Experiment 2: Impact on aposymbiotic juveniles

For each replicate, ten newly hatched aposymbiotic juveniles (without symbiotic algae) were collected and exposed to 4 different *p*CO_2_ (from 390 to 27000 µatm) for up to 5 days (without the presence of the symbiont, juveniles die within 10 days, Bailly, pers. obs.) Mortality was monitored every day.

#### Experiment 3: Impact on the establishment of symbiosis

For each replicate, ten newly hatched juveniles (without symbiotic algae) were collected from the culture, measured and then exposed to 4 different *p*CO_2_ (from 390 to 27000 µatm). 200 µl of *T. convolutae* culture was then added. Mortality was checked daily for 9 days. At day 9, animals were anesthetized, measured (total length) and checked for the presence of the symbiotic algae. Growth rate was calculated as the difference in size between the beginning and end of the experiment, divided by the exposure time (9 days).

#### Experiment 4: Impact of fertility

For each replicate, ten adults were collected in the cultures and exposed to 4 different *p*CO_2_ (from 390 to 27000 µatm). At day 9, the number of cocoons and number of eggs per cocoon were counted.

#### Experiment 5: Impact on adult survival

For each replicate, ten adults were collected in the cultures and exposed to 6 different *p*CO_2_ (from 390 to 272000 µatm). Mortality was checked daily for 3 days and integrity of the symbiosis was visually determined under binocular microscope by the presence of green algae with the worm.

### Statistics

Logarithmic regression model were used to test the relationship type between the measured parameters and CO_2_ concentration. The Shapiro-Wilk test [46] was used to confirm that the data were normally distributed and the Levene test was used to confirm that variances were homogenous. All data were analyzed using SAS/STAT software.
